# Annual Meeting of the Michigan Dental Association

**Published:** 1865-02

**Authors:** G. W. Stone


### ANNUAL MEETING OF THE MICHIGAN DENTAL ASSOCIATION.

YPSILANTI, June 10th, 1865.

The Tenth Annual Meeting of the Michigan Dental Association was held at the Hawkins House at 9, A. M.

The President, Dr. STONE, in the Chair.

Minutes of last meeting read and approved.

Minutes of special meeting read and approved.

Constitution read by Dr. Metcalf, of Kalamazoo.

The following named persons were examined and admitted as members of the Association, to wit: Dr. B. Banister, Kalamazoo; J. H. Warner, of St. Clair; Dr. North, of Ann Arbor; H. C. Phillips, of Coldwater; G. C. Taylor, Monroe.

Committee on Essays reported and were discharged. Report accepted.

The following officers were elected for the ensuing year, to wit:

Dr. J. A. WATLING, of Ypsilanti, President.
" G. W. FIELD, Detroit, Vice "
" G. W. STONE, Albion, Secretary.
" H. BENEDICT, Detroit, Treasurer.

Drs. ROBINSON, DOUGLAS, and METCALF, Examining Executive and Publishing Committee.

The President elect, on being conducted to the chair by Drs. Metcalf and Cahoon, made a few appropriate remarks. Dr. Stone, on retiring from the chair, delivered an interesting address, which, on motion, was referred to the publishing committee. Adjourned to 1½ P. M.

G. H. MOSHER, Secretary.

AFTERNOON SESSION.

Meeting called to order by the President, Dr. Watling. Report of Committee on Mechanical Dentistry called for. No report made. Report of Committee on Extracting called. No report made. Subject open for discussion.

Dr. Robinson extracts few teeth. Related a case in his own family. Boy 16 years old. Extracted the 2d molar—tooth healthy except slight inflammation of palatine root—first molar formerly filled—no soreness—thinks 2d molar could have been saved with proper treatment—recommends saving the deciduous teeth their full time—regulates them if necessary instead of extracting.

Dr. Cahoon would have given cathartics and a wash of black pepper tea to Dr. Robinson's boy instead of extracting his tooth.

Dr. Geary spoke of a similar case to that of Dr. Robinson's boy.—Extracts few teeth—uses lancet for depleting inflamed gums.

Dr. Metcalf uses the turnkey in extracting superior bicuspids—turns them in.

Committee on alveolar abscess made a verbal report.

Dr. Benedict related a case of abscess at apex of superior lateral incisor, punctured alveolus. Syringed with water, iodine and creosote.

Dr. Cahoon never fails of success when there is vitality in the tooth—punctures to the apex of palatine root of molars—treats the system.

Dr. Douglas related a case of a girl eight years old—abscess at apex of cuspid—discharged through cheek half way between the wing of nose and the eye—hole through process into antrum—roots of bicuspids absorbed—roots of incisors diseased. Syringed with tinct. myrrh, chlorate of soda—bone appeared green and tough. Treated the case for fifty successive days. Patient's health otherwise good—no bad taste in mouth—thinks it was necrosis.

Dr. Cahoon thinks there was much patience exercised in treatment of the case—thinks the dead bone should have been immediately cut away.

Dr. Gerry thinks "on authority," that the dead bone should not be cut away until a distinct line is formed between the dead and living bone.

Dr. Metcalf thinks Dr. Douglas exercised much patience, but says necrosis will never cure itself—has seen many similar cases—removed all dead matter immediately.

Dr. Bancroft was told by the girl treated by Dr. Douglas that she fell on a chair when picking cherries, and hurt her cheek bone.

Dr. Cahoon related a case of abscess at apex of superior lateral cavity as large as a pea—broke down dead bone with a chisel—injected iodine—cured in a short time.

Dr. Watling assures patients of success in his treatment—punctures at apex of fang.

Dr. Benedict spoke of models exhibited at the American Dental Association at Niagara Falls, by Dr. Pease of Dayton, Ohio, of similar cases to that of Dr. Cahoon.

On motion, an order of five dollars was drawn to pay the landlord of the Hawkins House for use of room.

On motion, adjourned to meet at the Follet House at 7 o'clock, P. M.

EVENING SESSION.

President in the Chair. Fitting Artificial Teeth was taken up.

Dr. Rix used to have some trouble with plates rocking—to prevent which he now scrapes off the alveolar ridge of model—succeeds well. Much discussion followed. Some contending for a well-defined air chamber, and others denouncing it as a nuisance.

Dr. Field takes a wax impression first, strikes a plate, perforates and uses it as an impression cap. Fills in part, and then crowds in an old model that is done swelling.

Dr. Benedict has some trouble and expects to have.

Dr. Mosher exhibited some burs made of wooden spools dipped in glue and emeried; some coarse and some fine.

Dr. Robinson called the attention of the association to the propriety memorializing the regents of the University for the establishment of a Dental Chair in said institution,. Some remarks mere made by Dr. Watling and others. On motion, it was resolved that a committee of five be appointed to confer with said Regents in regard to the feasibility of establishing such chair. Committee consisted of Drs. Robinson, Field, Cahoon, Porter and Benedict.

Adjourned till to-morrow morning at 9 o'clock.

MORNING SESSION.

Called to order by the President.

Dr. Mosher presented a bill of $18.96 for expenditures as past Secretary, and, on motion, it was ordered paid.

Dr. Porter submitted a report, as past Treasurer, showing a balance on hand of $138.75. On motion, the report was adopted.

On motion of Dr. Porter, it was resolved, that when this Association adjourns it adjourn to meet in the City of Detroit on the last Tuesday in January, 1866, at —— o'clock.

On motion, Drs. Field Bancroft, Gerry, Harris, Douglas, and Robinson were elected representatives to the American Dental Association.

Dr. Porter read a paper on Mechanical Dentistry. In which he ignored the term Mechanical Dentistry, Issue was taken, and much discussion followed.

Dr. Cahoon thanks the Lord that he is a mechanic; he likes the term.

Dr. Porter thinks the manufacturers of dental materials are the mechanics, but ignores the term as applied to dentistry.

On motion, the paper was referred to the publishing committee.

On motion, the subject of filling teeth was taken up, and the first order of business for the afternoon session shall be clinic operations.

Dr. Metcalf made some remarks and introduced a nicely arranged apparatus for annealing gold foil, which was favorably received by the Association.

On motion, Dr. Watling was requested to fill a tooth with the mallet, whereupon the Association was invited to meet at his office. Adjourned till 1½ o'clock.

AFTERNOON SESSION.

Operations by Drs. Watling, Benedict, Cahoon and others, using the mallet, and Dr. Metcalf's "Annealing Lamp."

Adjourned to 7 o'clock.

EVENING SESSION.

President in the Chair.

On motion, the Secretary was instructed to notify the members of the Association that the next annual meeting would last three days.

On motion of Dr. Robinson, the following resolution was adopted, to wit:

*Resolved*, That it is the duty of every member of this Association to furnish at the next regular meeting an essay on some subject connected with the profession that shall be submitted to the committee on publication to be circulated among the members of this Association.

Dr. Robinson presented also the following resolution, and on motion, it was adopted:

*Resolved*, That the "Annealing Lamp" exhibited to this Association, and invented by Dr. Metcalf, of Kalamazoo, for annealing gold foil, is an apparatus worthy the attention and patronage of every member of the dental profession.

The following resolution was presented by Dr. Metcalf, and, on motion, it was adopted:

*Resolved*, That this Association present to the member who shall construct the best automaton mallet for plugging teeth, a suitable medal worth at least twenty-five dollars, and in case there is only one offered it shall be preferable to any one in market.

On motion, the resolution of Dr. Robinson in regard to essays, shall be printed on the cards of invitation. Professional fees was taken up for discussion, in which nearly all present participated. It was generally considered that fees were regulated by professional standing.

On motion, adjourned. G. W. Stone, Secretary.

